# Autologous Chondrocyte Implantation on Polyethersulfone Scaffolds in a Rabbit Model of Grade III Lesions

**DOI:** 10.3390/molecules31081302

**Published:** 2026-04-16

**Authors:** Maciej Płończak, Monika Wasyłeczko, Tomasz Jakutowicz, Andrzej Chwojnowski, Jarosław Czubak

**Affiliations:** 1 Mazovia Regional Hospital John Paul II, 08-110 Siedlce, Poland; 2 Nałęcz Institute of Biocybernetic and Biomedical Engineering, Polish Academy of Sciences, 02-109 Warsaw, Poland; mwasyleczko@ibib.waw.pl (M.W.); achwojnowski@ibib.waw.pl (A.C.); 3 Department of Pediatric Orthopedics and Traumatology, Medical University of Warsaw, 02-091 Warsaw, Poland; jakutowicztom@gmail.com; 4 Department of Orthopedics, Pediatric Orthopedics and Traumatology, Centre of Postgraduate Medical Education, Gruca Orthopaedic and Trauma Teaching Hospital, 05-402 Otwock, Poland; czubakjarek@gmail.com

**Keywords:** 3D scaffolds, polyethersulfone (PES), autologous chondrocytes, articular cartilage, cartilage tissue engineering, preclinical rabbit model

## Abstract

Articular cartilage has a limited capacity for self-repair, and effective strategies for its regeneration remain a major clinical challenge. Full-thickness cartilage defects extending to the subchondral bone induce an enhanced inflammatory response and impair spontaneous healing. This study aimed to evaluate the regenerative potential of autologous chondrocyte transplantation using an insoluble polyethersulfone (PES) scaffold in a rabbit model of grade III articular cartilage lesions. Chondrocytes were isolated and expanded in vitro and subsequently seeded onto PES membranes. Sixty-two rabbit knees with defects extending to the subchondral bone were divided into three groups: group I received chondrocyte-seeded PES scaffolds (n = 25), group II received cell-free PES scaffolds (n = 25), and group III served as an untreated control (n = 12). Cartilage regeneration was evaluated macroscopically and histologically over 52 weeks. In addition, the chondrogenic differentiation potential of cells cultured on PES scaffolds was assessed. This study extends our previous investigations of PES scaffolds in grade IV cartilage defects to a clinically relevant grade III lesion model, enabling evaluation of regenerative outcomes at an earlier stage of cartilage degeneration. The results demonstrated superior tissue regeneration in defects treated with chondrocyte-seeded PES scaffolds compared to both control groups. These findings indicate that synthetic PES scaffolds support cartilage repair and represent a promising biomaterial for the development of cell-based therapies in articular cartilage regeneration.

## 1. Introduction

Hyaline cartilage covers the ends of bones, enables low-friction movement of joint surfaces under mechanical load, and ensures proper joint function. These properties result from its unique biomechanical characteristics, including low friction, resistance to compression, hardness, and durability [1,2,3,4,5,6].

A layer of calcified cartilage separates the articular cartilage from the bone marrow and blood vessels, which actively participate in generating the inflammatory response to injury. Therefore, it is important to determine whether the damage is limited to the cartilage itself or penetrates deeper into the subchondral bone layer [1,2,3,4].

Damage limited to the cartilage layer does not induce an inflammatory response due to the absence of bone marrow-derived cells, resulting in a lack of spontaneous healing. Chondrocytes surrounding the defect exhibit limited proliferation and matrix production and do not migrate into the damaged area, preventing effective regeneration even in small defects [6,7,8]. In contrast, progressive cartilage degradation, as observed in degenerative joint disease, leads to chondrocyte death, cartilage fragmentation, and gradual exposure of the subchondral bone [9,10,11,12,13].

Pain and impaired joint function resulting from articular cartilage damage have prompted the development of various treatment strategies. Despite significant advances, predicting the quality, quantity, and mechanical properties of regenerated tissue remains challenging. The primary objective of cartilage repair is to restore the articular surface with tissue resembling native hyaline cartilage to delay the progression of degenerative disease [12,14,15,16,17].

Bone marrow stimulation techniques, such as microfracture and microdrilling, are commonly used to treat cartilage defects. These methods aim to induce a repair response by enabling bone marrow-derived cells to migrate into the defect site [18,19,20,21,22]. Although initial formation of hyaline-like cartilage may occur, the regenerated tissue often undergoes fibrotic transformation over time, resulting in fibrocartilage with inferior biomechanical properties [6,22,23].

Another therapeutic approach involves osteochondral grafting, in which damaged cartilage is replaced with auto- or allogeneic hyaline cartilage supported by a bone substrate. Techniques such as mosaicplasty allow for the reconstruction of load-bearing joint surfaces using osteochondral cylinders harvested from non-weight-bearing regions [6,24].

Autologous chondrocyte implantation (ACI) represents an alternative method for introducing a new population of cells into cartilage defects. This two-stage procedure involves harvesting cartilage from a non-weight-bearing area, in vitro expansion of isolated chondrocytes, and subsequent implantation of the bioengineered construct into the defect site. Despite high costs and prolonged rehabilitation, the clinical effectiveness of ACI has been confirmed in numerous studies [6,24,25,26,27,28,29].

In cartilage tissue engineering, scaffolds are used to support the spatial organization of chondrocytes, protect developing tissue, and provide an appropriate environment for extracellular matrix formation. Scaffold materials must be biocompatible, mechanically stable, and capable of maintaining their structure under physiological conditions [6,30,31,32,33,34,35,36,37].

Natural polymers, such as collagen [38,39,40], hyaluronic acid (HA) [41,42], chondroitin sulfate (CS) [43,44], or fibrin [30,45,46], exhibit excellent biocompatibility and promote cell adhesion and extracellular matrix production. However, their rapid degradation and limited mechanical stability restrict their long-term effectiveness as cartilage scaffolds [6,30,31,32,33,38,47,48]. Synthetic polymers, including polycaprolactone (PCL) [49,50,51,52], polylactic acid (PLA) [53,54,55,56], and polyethersulfone (PES) [6,56,57,58,59,60,61,62,63,64,65], present a more diverse and promising array compared to natural counterparts. These polymers enable the fabrication of diverse membrane structures using various techniques and offer favorable physical, chemical, and mechanical properties [6,64]. Many synthetic polymers degrade into non-toxic components within the body. Hybrid scaffolds combining natural and synthetic polymers further enhance mechanical performance and biofunctionality [6,31,32,33,34,35,38,48,62,66,67,68,69,70,71,72,73,74,75,76,77,78].

Despite extensive preclinical and clinical research, most commercially available scaffolds for cartilage regeneration are based on natural polymers, primarily collagen. Due to their limited mechanical stability and rapid hydrolysis, these materials often fail to support the formation of durable hyaline cartilage, resulting instead in fibrocartilage tissue prone to degeneration [6,31,32,33,47,79,80]. Therefore, effective cartilage regeneration remains a challenge, highlighting the potential of three-dimensional synthetic scaffolds for joint surface reconstruction [6,15,16].

Polyethersulfone (PES) is a synthetic polymer characterized by high biostability, chemical and mechanical resistance, and surface modification potential, making it an attractive candidate for cartilage tissue engineering applications. Its use offers an alternative to natural scaffolds with limited durability and variable biological performance. Although PES is considered a non-degradable polymer under physiological conditions, previous in vivo studies in rabbit models [57,65] and in vitro experiments in simulated body fluid (SBF) have demonstrated partial scaffold mass reduction over time [62].

Previous studies have demonstrated the potential of PES-based scaffolds for cartilage tissue engineering. Blends of PES with other polymers, such as polyurethane, have been investigated primarily to evaluate their physicochemical properties and degradation behavior, indicating the possibility of modifying scaffold performance for biomedical applications [62]. Experimental in vivo investigations using rabbit models confirmed that autologous chondrocytes implanted on PES scaffolds promoted the formation of repair tissue with cartilage-like morphology and good integration with surrounding tissue [65]. Comparative studies evaluating autogenic and allogenic chondrocyte transplantation on PES scaffolds further demonstrated that the polymer provides favorable structural support for cell delivery and tissue formation, while also highlighting limitations related to incomplete tissue maturation [57].

Previous experimental studies have also shown that PES scaffolds support chondrocyte viability and facilitate the formation of cartilage-like repair tissue, while comparative investigations highlighted differences in tissue maturation between autogenic and allogenic cell sources [56,57,62,64,65]. These findings provide context for the structural and biological behavior of PES scaffolds and help justify their continued evaluation in early-stage cartilage defects.

In our previous study, polyethersulfone (PES) scaffolds were investigated in a rabbit model of grade IV cartilage defects; the present work extends these findings by evaluating the regenerative potential of PES-supported autologous chondrocyte implantation in less advanced, clinically relevant grade III lesions.

This study aimed to evaluate the regenerative effect of autologous chondrocyte implantation on polyethersulfone (PES) scaffolds in a rabbit model of grade III articular cartilage defects extending to the subchondral bone. An efficient protocol for chondrocyte isolation and in vitro culture was developed. The regenerative process was assessed macroscopically and histologically over 52 weeks, and the chondrogenic capacity of cells cultured on non-degradable PES membranes was evaluated.

## 2. Results and Discussion

### 2.1. Morphological Characterization of PES Scaffold

**Figure 1:**
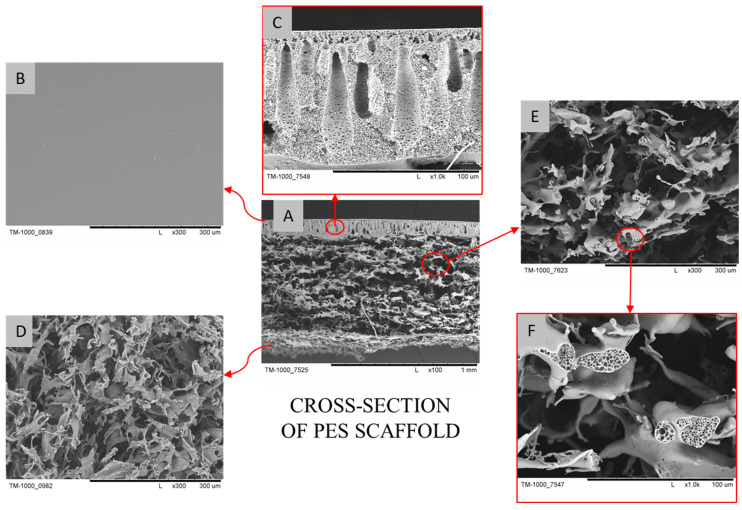
The SEM images of the PES scaffold: (**A**)—cross-section; (**B**)—dense bottom layer; (**C**)—magnified view of the bottom layer with micropores; (**D**)—perforated top layer; (**E**)—magnified view of the scaffold cross-section; (**F**)—micropores in the scaffold cross-section. Scale bars: (**A**)—1 mm; (**B**,**D**,**E**)—300 µm; (**C**,**F**)—100 µm.

The morphology of the PES scaffold was evaluated using scanning electron microscopy (SEM). As shown in Figure 1, the surface (top layer) of the PES scaffold is porous, facilitating cellular infiltration into the scaffold. The bottom layer is smooth and compact, preventing cells and their products from escaping the scaffold. The walls of the scaffold are microporous, allowing for the delivery of oxygen and nutrients to the cells and the removal of metabolites. Additionally, the epidermal layer on the top surface of the scaffold was removed, which further increased the porosity of the top layer and enhanced the potential for cell penetration into the interior of the PES scaffold.

The PES polymer is classified as a non-degradable high-performance thermoplastic polymer [64]. Nevertheless, previous in vivo studies in rabbit models [57,65] have reported evidence of PES scaffold degradation over time, indicating that minor structural changes may occur under physiological conditions. Additionally, in vitro experiments using simulated body fluid (SBF) demonstrated a measurable reduction in scaffold mass [62], further supporting the notion that PES is not completely inert and can undergo limited degradation under specific conditions. These observations provide valuable context for interpreting the long-term persistence and integration of the scaffold in vivo. While the scaffolds in the present study maintained their overall architecture, the potential for minor degradation highlights the importance of ongoing monitoring in future investigations to fully understand scaffold–tissue interactions and their implications for cartilage repair.

### 2.2. Number of Cells and Observation of Chondrocytes

Chondrocytes were isolated enzymatically and subsequently counted using a Bürker chamber. Each isolation yielded approximately 1–1.5 × 10^5^ viable cells. An identical number of cells was inoculated onto PES scaffolds positioned in six-well culture plates.

**Figure 2:**
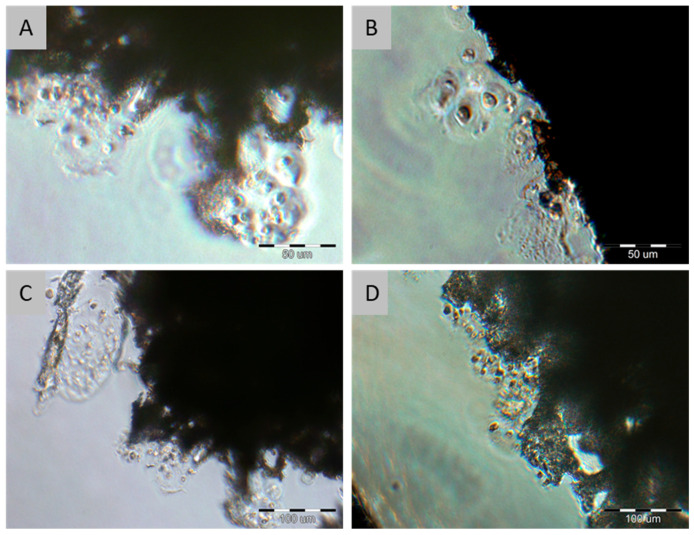
Chondrocytes on the edges of the PES scaffolds after 14 days of culture. Scale bars: (**A**,**B**)—50 µm; (**C**,**D**)—100 µm.

After 14 days of in vitro culture, cell morphology and distribution were assessed using an inverted microscope. Figure 2 shows chondrocytes adhering to the peripheral regions of the scaffold.

**Figure 3:**
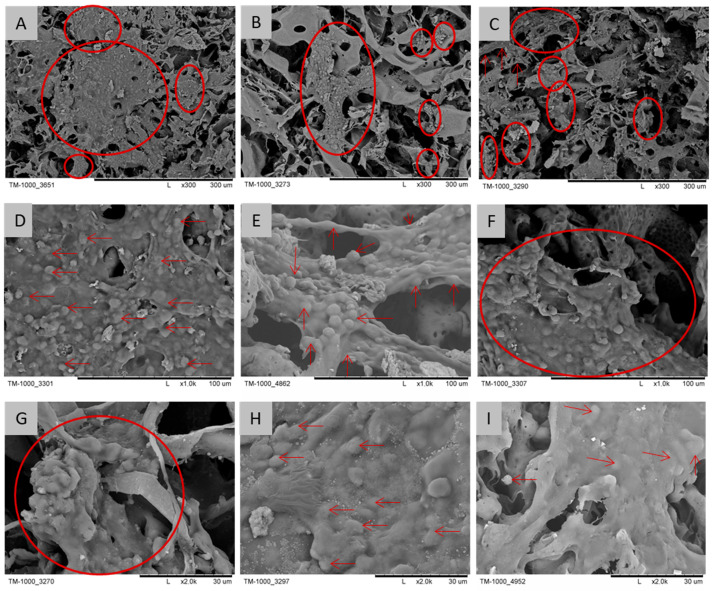
SEM images of the PES scaffold after 4 weeks of cultivation, with red arrows and circles marking the extracellular matrix and chondrocytes. Scale bars: (**A**–**C**)—300 µm; (**D**–**F**)—100 µm; (**G**–**I**)—30 µm.

The presence and surface attachment of cells on the PES scaffolds were further confirmed by scanning electron microscopy (SEM) (Figure 3). SEM analysis demonstrated that the cells were incorporated into the scaffold surface and enveloped by extracellular matrix structures, as highlighted by red circles and arrows.

The isolated and in vitro-expanded cells expressing the gene for type II B collagen—a molecular marker characteristic of cartilage tissue [81]—display metabolic features typical of chondrocytes. At the same time, the presence of type I collagen gene expression, typical of fibroblasts, suggests dedifferentiation within the cell culture, resulting in a phenotype resembling mesenchymal prechondrogenic cells with fibroblast-like morphology [82]. According to some authors, culturing cartilage cells at the bottom of a culture flask causes their irreversible dedifferentiation and cessation of type II collagen and proteoglycan production [8]. Wang Y. et al. [83] demonstrated that electrospun three-dimensional structures exhibit excellent biocompatibility, appropriate porosity, and the ability to support and differentiate cartilage cells. Their potential as functional scaffolds in regenerative orthopedics was also emphasized. The PES scaffold offers a three-dimensional spatial environment that better supports chondrocyte proliferation compared to conventional two-dimensional monolayer cultures. PES scaffold is a biocompatible material used in medicine, including dialysis. Its unique structure provides excellent conditions for newly formed cartilage tissue. The PES scaffold has micropores measuring 0.01–3 µm, enabling the transport of nutrients and the removal of metabolites from cartilage cells that inhabit macropores with diameters of 40–500 µm. It can be assumed that cartilage cells in the conditions created by the porous sulfone polymer undergo less dedifferentiation. Earlier research has shown that the scaffold is suitable for supporting the culture of both rabbit chondrocytes [57,65] and human chondrocytes [56]. Cell scaffolds with porosity in the range of 65–90% and pore diameters of 50–400 µm exhibit parameters considered optimal for cell growth and differentiation in the literature, as confirmed by, among others, Lin X. et al. [84]. Chen et al. [85] and Wu X. et al. [86] indicate in their articles that appropriate porosity and a network of interconnected pores are essential for effective cell migration, nutrient diffusion, and metabolite removal. Structures of this type promote vascularization and effective tissue regeneration. It should be noted that the optimal pore size depends on the cell type; while the range used in the present study is suitable for chondrocytes, other cell types, such as bone cells or adipose-derived stem cells, may require larger pores to support growth and differentiation [87,88]. An important complement to our own data are literature reports [57,64], which confirm the potential of the PES polymer as a biomedical material, and the studies presented in the article by Jakutowicz et al., which showed that PES scaffolds have comparable regenerative effectiveness when using both autologous and allogeneic chondrocytes, which opens up prospects for the development of therapies based on allogeneic cell transplants.

A prior RT-PCR analysis conducted on an animal model revealed the expression of type II procollagen, characteristic of hyaline cartilage [65]. In our previous studies, the expression of the gene encoding procollagen type II, a molecular marker of chondrocytes, was evaluated in cells cultured on the PES membranes. These data indicate the expression of type II procollagen without exon 2 (type IIB collagen), which is characteristic of cartilage. The gene expression analysis confirmed the presence of cartilage-specific markers, including collagen type II, indicating chondrogenic activity within the scaffold environment.

### 2.3. Elemental Analysis

The PES scaffold slices after 14 days of cultivation with chondrocytes showed a concentration of approximately 0.24 ± 0.035 mg of tissue in one part of the scaffold (Table 1).

Elemental analysis of membranes retrieved from the culture vessel two weeks after culture initiation confirmed tissue formation on the PES scaffold. The detection of approximately 0.24 mg of tissue on a single 0.5 cm diameter sample suggests that the PES polymer offers a favorable microenvironment for tissue development. In our previous in vitro studies, we observed an increase in protein expression in human chondrocytes cultured on PES scaffolds, supporting the conclusion that the polymer provides a supportive microenvironment for cell growth [56].

This three-dimensional scaffold structure enables unrestricted tissue growth, potentially limiting the tendency of chondrocytes to adopt a fibroblast-like morphology, which is commonly observed in traditional monolayer cultures at the bottom of the culture plate.

### 2.4. Macroscopic Assessment

At 12, 28, and 54 weeks post surgery, the treated knee joints preserved their normal anatomical shape and demonstrated complete mobility.

#### 2.4.1. Degree of Defect Repair

**Figure 4:**
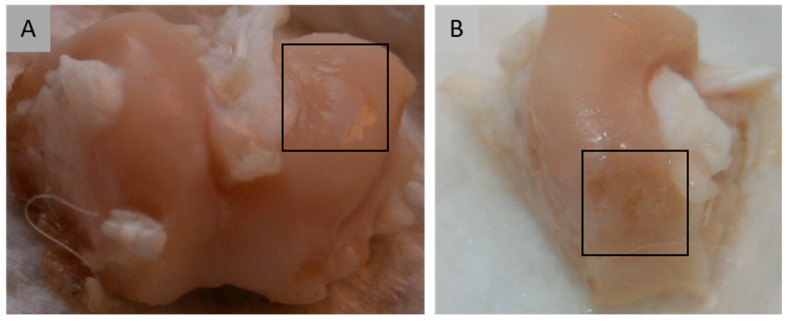
Macroscopic assessment of the knee cartilage defect after 52 weeks: (**A**)—group I: the defect was completely filled with newly formed tissue; (**B**)—group III: the defect showed minimal cartilage filling.

In almost 90% of cases in group I, the filling of defects ranged from 75% to 100%, indicating significant spontaneous regeneration after 52 weeks (Figure 4).

According to the ICRS scale [82], every group of rabbits was subsequently analyzed and categorized into subgroups according to the scores obtained. After 12 weeks of the surgery, 1/4 of the defects were filled with cartilage-like tissue, after 28 weeks, 1/3, and after 54 weeks, almost half. In group II, after 12 and 28 weeks, more than 75% of the defect was filled in 25% of cases, and after 54 weeks in 78%. In group III, in all operated rabbits, the degree of filling of the defects did not exceed 50%.

The average ratings in group I rabbits were 3.2 in all observation periods; in group II, in the first two periods, the average of 2.1 increased to 3 in the longest period. Therefore, complete replacement of defects was observed and statistically confirmed in the largest number of operated joints in group I rabbits. The lowest results were characteristic of group III, in which regeneration occurred only to a small extent. We did not find statistically significant differences between the assessments in individual observation periods, except for group II, in which, after a year, the number of joints regenerated by 75 to 100% approached the level obtained in group I.

The most extensive defect filling was observed and statistically confirmed in Group I, where the highest number of operated joints demonstrated substantial tissue regeneration. In contrast, group III exhibited the lowest repair outcomes, with only minimal regeneration detected. No statistically significant differences were observed between time points within the individual groups, except for group II. In this group, after 52 weeks, the number of joints with 75–100% defect filling increased and approached the level observed in group I.

#### 2.4.2. Macroscopic Appearance

A total of 20% of the regenerates in group I and 8% in group II had an articular surface similar in appearance to the surrounding healthy tissue and were rated the highest. In group III, the regenerated articular surfaces had numerous pores or were completely disintegrated.

The average result in group I was 2.9, in group II it was 2.2, and in group III it was 0.6.

There were no statistically significant differences between groups I and II, and the distribution of results was similar in all observation periods. Comparing groups I and II in terms of the macroscopic appearance of the regenerate surface, there was a slight difference between the group in which cells were transplanted (I) and the group in which only a PES scaffold was transplanted (II). Comparing both the means and the percentage of results describing smooth and uniform cartilage surfaces, we see an advantage of group I over group II, although this was also not statistically confirmed. In group III, the regenerated joint surfaces, which had numerous gaps or were completely disintegrated, received significantly lower scores.

#### 2.4.3. Summary of Results in Macroscopic Assessment

A total of 85% of the regenerates in group I and 36% in group II received the highest ratings, scoring between 6 and 8 points (Table 2). In group III, the lowest rated regenerates accounted for 41% of the results.

The average of all grades in group I was 6.1, in group II it was 4.6, and in group III it was 1.7 (Table 3).

A statistically significant difference was found between the number of points obtained in groups I and II in the shortest and medium observation periods. There were no statistically significant differences between individual observation periods (Table 4). Group III was not included in the statistical analysis because the group was too small. In group I, where cartilage cells were introduced into the defects on a scaffold, better tissue regeneration was achieved in a shorter time compared to group II, where only an empty scaffold was introduced. It is possible that the presence of a multiplied number of chondrocytes as a result of tissue culture increases the dynamics of the healing process of defects in the cartilage surface, and then, after a longer observation period, the regeneration results become similar in both analyzed groups (Table 5).

### 2.5. Microscopic Assessment

Histological evaluation was performed using a semi-quantitative scoring system commonly used in cartilage repair studies. Microscopic assessment was conducted independently for each experimental group, with tissue samples evaluated according to the O’Driscoll histological scoring system [89,90]. This standardized grading method was used to assign numerical scores reflecting the quality of cartilage repair. Based on the total number of points obtained, samples were further classified into subgroups corresponding to different levels of regenerative response.

#### 2.5.1. Nature of Predominant Tissue

In groups I and II of the assessed regenerates, tissue similar in properties to hyaline-like cartilage tissue prevailed, accounting for 72% and 68% of the results.

The mean results in groups I and II were 3.4 and 3.2, respectively. In group III, regenerates composed of fibrocartilage predominated, accounting for 56%; the average result was 1.

**Figure 5:**
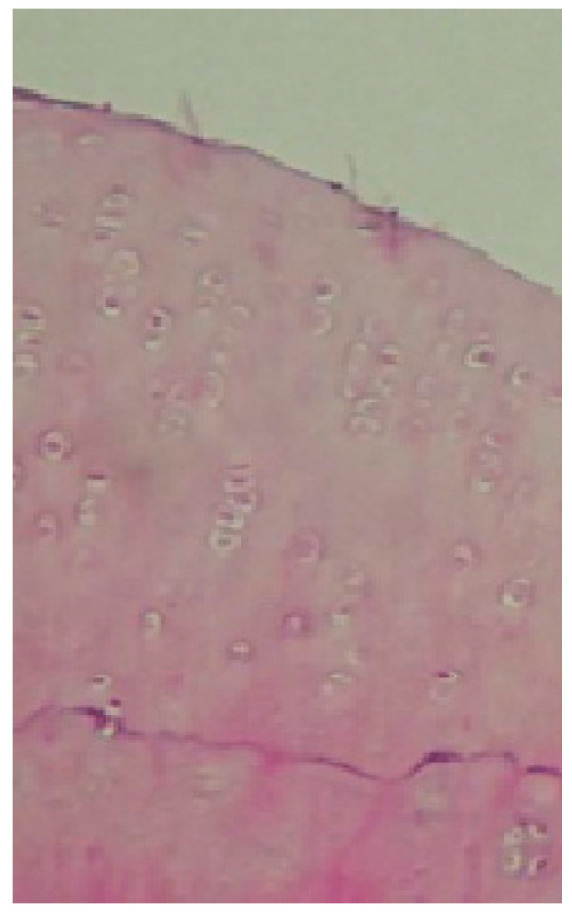
Representative histological images of defect repair in rabbit articular cartilage: group I, the defect is filled with tissue resembling mature hyaline-like cartilage, characterized by a homogeneous matrix structure and chondrocyte-like cells embedded in lacunae (magnification 200×).

To illustrate the histological differences in tissue regeneration between experimental groups, representative microscopic images were selected (Figure 5). These images highlight the quality of defect filling and the type of tissue formed in the most (hyaline-like cartilage) and least (fibrocartilage) successful repair cases, as observed in groups I and III.

There were no statistically significant differences between groups I and II.

The distribution of scores in groups I, II, and III did not change significantly with time after the procedure.

#### 2.5.2. Structural Characteristics

##### Surface Regularity

In groups I and II, the surface of the regenerates after 12 weeks was irregular; 75% of the grades in group I and nearly 90% in group II were regenerates with significantly or partially torn surfaces. Over longer periods of observation, the regularity of the surface gradually improved, and in the third observation period, nearly 90% of the regenerates in group I and 80% of the regenerates in group II had a smooth or slightly uneven surface.

**Figure 6:**
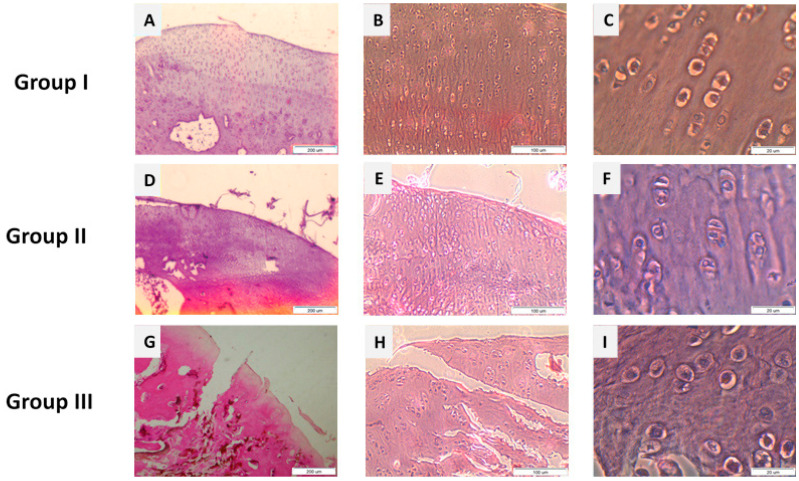
Microscopic images of histological samples from the studied groups. Images (**A**–**C**): group I after 52 weeks of observation. Images (**D**–**F**): group II after 52 weeks of observation. Images (**G**–**I**): group III (control group) after 52 weeks of observation. Scale bars: (**A**,**D**,**G**)—200 μm; (**B**,**E**,**H**)—100 μm; (**C**,**F**,**I**)—20 μm.

The average results in groups I and II in all observation periods were 1.5 and 1.2, respectively. In group III, the surface of the regenerates was 90% uneven and torn (Figure 6). The mean of the results in group III was 1.

**Figure 7:**
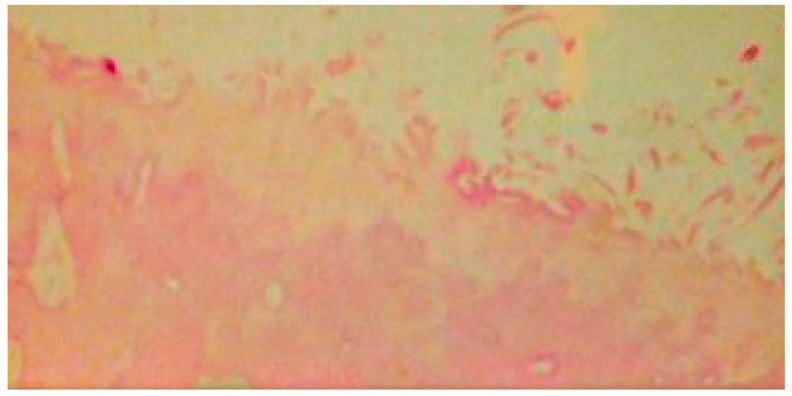
Representative histological image of regenerated cartilage in group III. The defect surface appears highly irregular, showing numerous fissures and discontinuities, which reflect the weak structural organization of the reparative tissue (magnification 200×).

In group III, the surface of the regenerates was irregular and frequently disrupted in the majority of samples, confirming the predominance of incomplete or disorganized tissue repair (Figure 7).

No statistically significant differences were observed between groups I and II regarding the surface regularity of the regenerated tissue. However, in group I, the differences between the first and second, as well as between the first and third observation periods, were statistically significant. Similarly, in group II, a significant difference was noted between the first and third observation periods. In both groups, the regularity of the cartilage surface was not fully restored after 12 weeks of follow-up. In group I, with transplanted cartilage cells, a significant improvement in surface regularity was observed after 25 weeks, while in group II, after regeneration with an empty scaffold, a similar improvement in the quality of the regenerated surface occurred after 52 weeks. Improvements in surface regularity were observed in group I after a shorter follow-up period compared to group II.

##### Structural Integrity

In group I, in 40% of cases, the regenerates were described as fully structurally integrated, and in 40%, cysts and interruptions were observed. In group II, 60% of the regenerates were characterized by the presence of slight breaks and cysts; the remaining 40% were the lowest grades. In group III, 66% of the regenerates were completely disintegrated.

The assessment of structural integrity revealed the highest average score in group I (1.2), indicating more advanced tissue organization within the regenerated area. Group II demonstrated a lower mean score of 0.6, while group III showed minimal structural integrity, with an average score of 0.3. These results reflect progressively reduced quality of tissue architecture across the experimental groups.

No statistically significant differences in structural integrity were observed between groups I and II. However, in group II, a statistically significant difference (*p* ≤ 0.05) was identified between the first and final observation time points, indicating a time-dependent improvement in tissue organization.

##### Thickness

The distribution of tissue thickness within the defect area varied between experimental groups. In group I, over 30% of the defects were filled with tissue matching the level of the surrounding native cartilage (score 2), while in group II, this proportion was higher, reaching 45%. Additionally, in 40% of cases in group I and 30% in group II, the tissue filled more than half the depth of the defect (score 1). In contrast, group III exhibited the lowest repair quality, with 60% of the samples showing minimal tissue formation (score 0).

Quantitative evaluation of the average thickness scores further supported these observations. The mean score in group I was 1.0, in group II it was 1.1, and in group III it was only 0.4, indicating substantially less tissue regeneration in the untreated control group.

In the statistical analysis, no significant differences were found between groups I and II. A significant improvement in grades was found in groups I and II in the longest observation period compared to the shortest.

##### Bonding to the Adjacent Cartilage

A total of 45% of regenerates in group I and 8% in group II were completely integrated with the surrounding cartilage; 45% in group I and 75% in group II were partially integrated. The quality of the regenerated tissue in group III was significantly inferior compared to the other groups. More than 75% of the specimens demonstrated poor integration with the surrounding native cartilage, and the rest were completely disintegrated.

**Figure 8:**
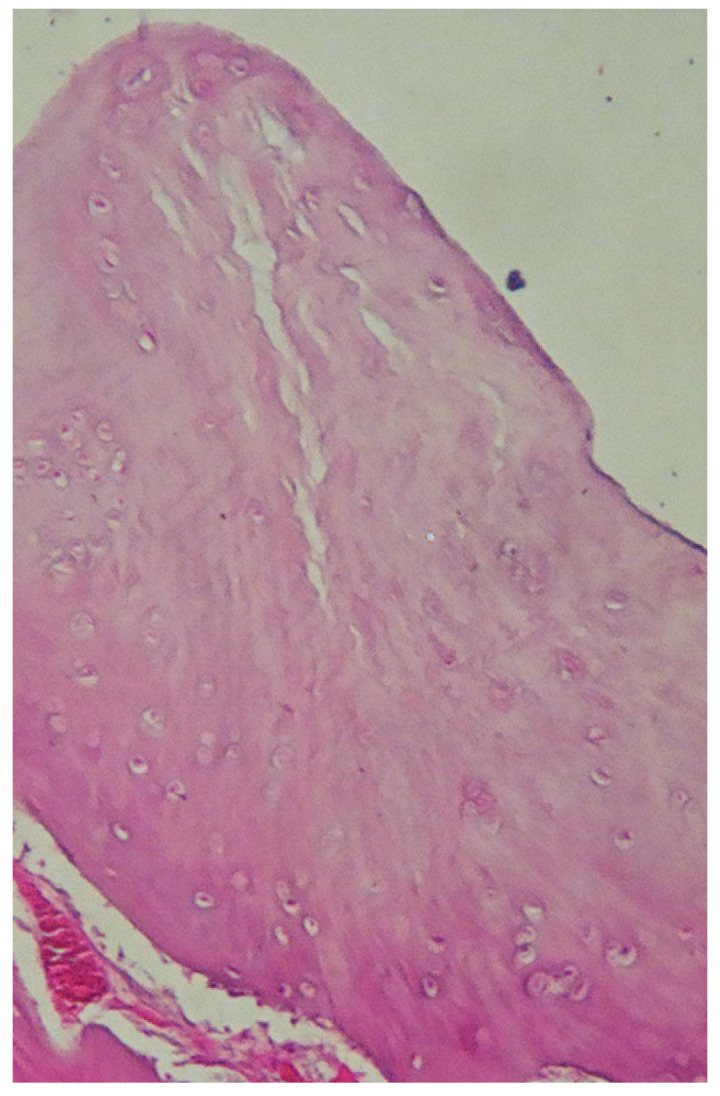
Representative histological image of the defect site in group III after 52 weeks. The regenerate is characterized by low cellularity and poor tissue organization. No evidence of hyaline-like cartilage is observed. Magnification 100×.

The average result in group I was 1.2, in group II it was 0.9, and in group III it was 0.6. Figure 8 shows the samples from each group after 52 weeks of observation. In groups I and II, the regenerated tissue surface is smooth and intact, with well-integrated cartilage and a thickness approximately equal to that of normal cartilage. In contrast, group III displays an irregular defect surface and poor tissue integration after 52 weeks.

Moreover, in the groups in which the PES scaffold was transplanted, there was no trace of the PES material.

There were no statistically significant differences in the assessments between groups I and II of rabbits and between individual observation periods.

##### Stage of Degenerative Cellular Changes—Hypocellularity

A total of 70% of the regenerated defects in group I and 56% in group II were filled with tissue exhibiting normal cellularity, whereas in group III, 66% of the defects received the lowest grades.

The average integration scores for groups I, II, and III were 2.3, 2.2, and 0.3, respectively.

To illustrate the long-term outcome of cartilage repair in the untreated control group, a representative histological image from group III at 52 weeks is presented (Figure 8). This example highlights the limited regenerative potential observed in the absence of scaffold-based intervention.

In the second group of rabbits, statistically significant differences were observed between the first and second, as well as the first and third, observation periods. There were no significant differences between groups I and II of rabbits.

In group I, with transplanted cartilage cells, normal cellularity was observed after 12 weeks, while in group II, after regeneration with an empty scaffold, normal cellularity occurred after 25 weeks, remaining stable up to 52 weeks of follow-up.

##### Stage of Degenerative Cellular Changes—Degenerative Changes

**Figure 9:**
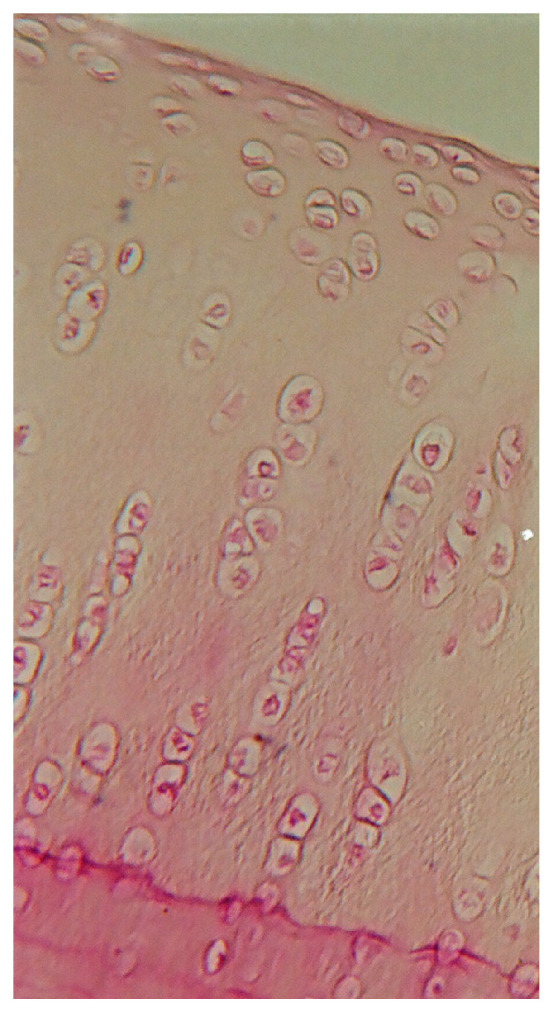
Representative histological image of regenerated cartilage in group I after 52 weeks. Viable chondrocytes are visible within the tissue, with no signs of necrosis or structural degradation. The repair tissue appears well organized. Magnification 100×.

In groups I and II, a small number of necrotic cells were found in the regenerates in the first two observation periods, and no low results were found in the third observation period. To demonstrate the quality of regenerated tissue in the scaffold-supported group, Figure 9 presents a representative histological image from Group I after 52 weeks. The image illustrates the presence of viable chondrocytes and the absence of necrotic cells within the repair tissue.

The distribution of degenerative cellular changes in groups I and II was as follows: No signs of necrosis were observed in 52% of the specimens, indicating relatively low levels of cellular degeneration in the majority of cases. In contrast, group III showed a markedly different pattern, with 60% of samples demonstrating low scores, reflecting more pronounced degenerative changes. Importantly, none of the regenerates in Group III were free of necrotic features. Histological analysis revealed the frequent presence of necrotic chondrocytes, intracellular vacuoles, and signs of edema within the repair tissue.

The average scores reflect the severity of degenerative cellular changes across groups. The mean score was 1.4 in both group I and group II, consistent with generally mild or absent degeneration. In contrast, group III exhibited a substantially lower average score of 0.4, confirming a high degree of cellular damage and poor regenerative quality in the absence of therapeutic intervention.

There were no significant differences between the assessments in groups I and II, and a statistically significant difference was found between the assessments in the shortest and the longest observation period in group II.

##### Comparison of the Total Scores Achieved Across the Different Rabbit Groups

Table 6 presents the mean total scores reflecting the overall quality of cartilage regeneration across experimental groups. In group I, the average sum of points was 12.5, indicating a generally favorable regenerative outcome. Group II demonstrated a slightly lower mean score of 11.5, while group III showed a markedly reduced regenerative capacity, with an average total score of only 4.5.

The detailed distribution of scores is shown in Table 7. In group I, 52% of the regenerated tissues scored in the highest range (15–18 points), and 25% fell within the moderate range (11–14 points). Only 12% of the specimens received the lowest scores. In Group II, 20% of the joints were rated in the 15–18 range, while the majority (48%) scored between 11 and 14 points; 12% were classified in the lowest range. In contrast, group III exhibited a significantly poorer outcome: 83% of regenerates were assigned the lowest scores (0–6 points), reflecting minimal tissue quality and insufficient repair.

No statistically significant differences were observed between the total scores of groups I and II (Table 8), indicating comparable overall outcomes in terms of cartilage regeneration between these two treatment groups.

However, analysis of score progression over time revealed statistically significant differences within group II (Table 9). A clear increase in the total scores was observed between the earliest and latest observation periods, suggesting that regenerative outcomes in group II improved with longer follow-up. No comparable temporal trend was identified in group I.

The group that received transplantation of in vitro-cultured cells placed on scaffolds showed significantly greater healing dynamics compared to the group that only had scaffolds in the cartilage defects. Over the 52-week follow-up period, the quality of the newly formed tissue became similar for both groups, significantly better than that of the group in which the healing process occurred spontaneously.

When comparing the microscopic images with the analogous images in the work of T. Jakutowicz and colleagues [57], the surface of regenerates in group I, in which the autogenic chondrocytes on PES scaffolds were transplanted into full-thickness defects (ICRS grade IV), after 12 weeks of observation, exhibited a smooth and intact surface, with the regenerated tissue reaching approximately the same thickness as normal cartilage. Group III, in which pure PES scaffolds were transplanted into the defective area (no chondrocyte transplants), showed that the surface of the regenerated tissue is smooth and the cartilage is well integrated and even achieved better outcomes in terms of microscopic evaluation over time (12 vs. 8 weeks postop). They assume that the improvement in microscopic evaluation over time in group III was due to the migration of the bone marrow stem cells into the scaffold. Similarly, in the S.R. Frenkel study, significantly better results of cartilage surface regeneration were obtained after implantation of an empty scaffold composed mainly of type I collagen compared to defects left by the natural healing process [91]. In group IV, where the lesions received no treatment (neither chondrocytes nor PES scaffolds were applied), the defect surface remained irregular and poorly integrated after 12 weeks of observation. Furthermore, in previous studies [57,65], in groups where PES scaffolds were transplanted, no traces of PES scaffolds were observed, suggesting their complete degradation before the end of the 12-week observation period.

### 2.6. Limitations

This study has several limitations that should be considered when interpreting the results. The assessment of outcomes was limited to semi-quantitative histological and macroscopic evaluations, without complementary molecular, biochemical (e.g., glycosaminoglycan content), or biomechanical analyses. Another limitation is the lack of validation of the tissue mass estimation method based on elemental analysis. In addition, the study was conducted on a relatively small number of animals, which reduces statistical power, although histological evaluation was performed by an independent blinded rater. Finally, this study was carried out exclusively on male rabbits, which limits the generalizability of the findings to both sexes.

Moreover, a limitation of this study is the lack of immunohistochemical characterization of cartilage-specific markers such as collagen type II, collagen type I, or aggrecan. In our previous studies involving PES scaffolds, gene expression analysis confirmed the presence of cartilage-specific markers, including collagen type II. However, due to financial constraints, such analyses were not performed in the present study. Therefore, the characterization of the regenerated tissue in this work is based primarily on morphological and histological observations.

## 3. Materials and Methods

### 3.1. Materials

#### 3.1.1. Scaffolds

The PES scaffold was designed and manufactured in cooperation with chemists from the Institute of Biocybernetics and Biomedical Engineering of the Polish Academy of Sciences in Warsaw. The choice of this material was determined by its favorable biomechanical properties, such as chemical resistance and the possibility of forming a membrane using easy methods. It was obtained via the wet inversion phase technique according to previous work [56,59,62,64,65,92]. Before cell seeding, the epidermal layer from the top surface of the scaffold was removed to enhance surface porosity and improve cell infiltration.

The scaffold preparation method and physicochemical characterization were described in detail in our previous work [56,57,59,65].

#### 3.1.2. Rabbits

**Figure 10:**
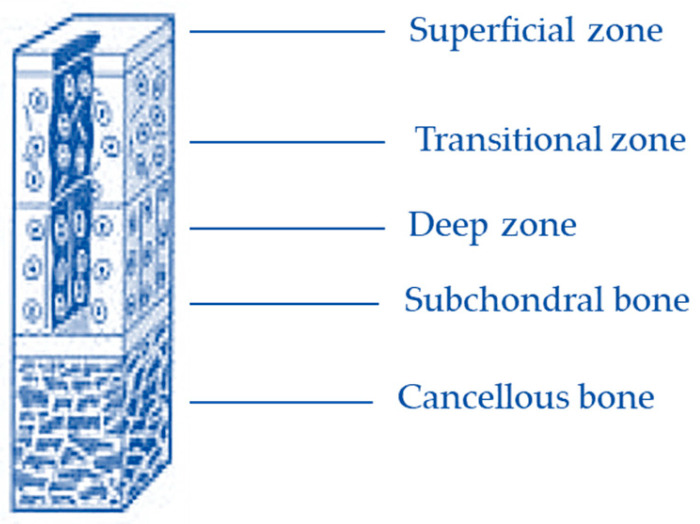
Loss of articular cartilage extending to the subchondral bone, corresponding to grade III cartilage injury as defined by the ICRS classification.

This study was conducted on the right or left knee joint of 62 White New Zealand male rabbits, each weighing between 2.0 and 3.5 kg and aged approximately 4 months. The animals were housed individually in standard laboratory conditions at the Miroslaw Mossakowski Institute of Experimental and Clinical Medicine, Polish Academy of Sciences, in Warsaw. Environmental parameters were maintained at a temperature of 24 ± 2 °C and a relative humidity of 50 ± 10%. Rabbits had unrestricted movement within their cages both before and after the surgical procedures. In all knee joints, standardized grade III defects were surgically induced on the articular surface (Figure 10).

Operational joints were divided into three groups:

- Defect reaching the subchondral bone, with chondrocytes placed on a PES scaffold, in 25 knees.
- Defect reaching the subchondral bone and implantation of a PES scaffold without cells, in 25 knees.
- Defect reaching the subchondral bone without any implant, in 12 knees.

The choice of the research model was determined by several considerations: the morphological and functional resemblance between rabbit and human articular cartilage; the relatively low cost of acquiring and maintaining the animals; the ease of administering general anesthesia in rabbits; the frequent use of this species in experimental studies on articular cartilage; and the availability of extensive research enabling reliable comparison with previously published findings. The study was approved by the First Warsaw Ethical Commission for Experiments on Animals of the M. Nencki Institute of Experimental Biology of the Polish Academy of Sciences in Warsaw, through Opinion No. 349/2004.

### 3.2. Methods

#### 3.2.1. Methods for Chondrocyte Isolation and Cultivation

In rabbits from group I, articular cartilage samples were obtained from non-weight-bearing areas of the joint surface. Tissue fragments were excised from the peripheral zones of the lateral and medial femoral condyles. Immediately after harvesting, the specimens were transferred into sterile tubes containing about 1.5 mL of physiological saline solution (0.9% NaCl) and transported to the laboratory for cell isolation and culture. The enzymatic digestion process was initiated within two hours of tissue retrieval. Under sterile conditions in a laminar flow chamber, cartilage samples were cut into ~1 mm slices using a No. 12 surgical blade. The tissue was subsequently washed multiple times with saline solution and placed into a sterile tube containing 0.25% collagenase type II in culture medium (RPMI enriched with DNase at 7.2 g/L [17.6 units/g], 10% fetal bovine serum (FBS), and 1.5% antibiotic mixture [streptomycin and penicillin, 100× dilution]). The mixture was incubated for 12 h at 37 °C in a 5% CO_2_ atmosphere with constant agitation. Following enzymatic digestion, the suspension was centrifuged at 1000 rpm for 5 min at 5 °C. The supernatant was discarded, and the resulting pellet was resuspended in 2 mL of culture medium. Before cell counting using a Bürker chamber (BRAND GMBH + CO KG (BLAUBRAND^®^), Wertheim, Germany), cells were stained with a 0.5% trypan blue solution to assess viability. Viable cells were seeded freely onto 5 mm diameter PES membranes placed in six-well culture plates, and supplemented medium was added to every well. The cells were cultured at 37 °C in an atmosphere containing 5% CO_2_. The implantation procedure was carried out 14 days following the biopsy.

#### 3.2.2. Procedure for Graft Implantation

All surgical interventions were performed under sterile conditions within the operating room. Rabbits from groups I and II underwent surgery under general anesthesia induced by intramuscular administration of ketamine hydrochloride (30 mg/kg) and xylazine (2 mg/kg). The animals were positioned on their backs, and after appropriate alignment of the limb, an anteromedial parapatellar arthrotomy of the knee joint was carried out. The patella was then carefully displaced laterally to provide access to the femoral trochlear surface.

A standardized cylindrical full-thickness chondral defect (5 mm in diameter, 2 mm deep) extending to the subchondral bone was created on the patellar groove of each femur. In groups I and II, the defect was filled with a polyethersulfone (PES) membrane, secured in place with 6-0 vicryl sutures. In group I, membranes were seeded with autologous chondrocytes, while in group II, acellular membranes were used. In group III (control group), the defect was left untreated. Closure of the joint capsule and skin was performed in layers using 4-0 vicryl sutures. Postoperatively, all animals were allowed unrestricted movement in individual cages, without the use of immobilization or external splints. Euthanasia was performed at 12, 25, and 52 weeks post surgery by administering an overdose of sodium phenobarbital.

#### 3.2.3. Elemental Composition Analysis

To determine the quantity of tissue developed on the PES membranes after cell culture, elemental composition analysis was performed at the Department of Analytical Chemistry, Faculty of Chemistry, Warsaw University of Technology. The protein on PES membrane disks (5 mm in diameter) was quantified based on their nitrogen (N) content. Analyses were conducted both before cell seeding (reference samples) and after a two-week chondrocyte culture period. After the culture period, the samples were fixed in 2.5% glutaraldehyde and then dried. Protein content was determined by multiplying the nitrogen concentration obtained from the analysis by the standard protein conversion factor of 6.25. In total, eight measurements were carried out—four for the reference membranes and four for the membranes following cell culture.

#### 3.2.4. Macroscopic Assessment

The extent of defect filling in relation to the adjacent healthy tissue and the surface smoothness was assessed macroscopically. For this evaluation, the International Cartilage Repair Society (ICRS) cartilage repair grading scale was applied [62,93]. Macroscopic assessment was performed by a single investigator blinded to group assignment.

#### 3.2.5. Microscopic Analysis

Decalcified material was embedded in Paraplast PLUS paraffin (Sigma Aldrich, Poznan, Poland). The blocks were cut in the frontal plane onto a slide using a microtome to a thickness of 4 μm. Paraffin sections were stained by using the routine hematoxylin–eosin method. Samples from several areas of regeneration were selected for microscopic evaluation. The reparative tissue was analyzed histologically. The cell morphology, regularity of surface, integrity of the structure, thickness, integration with surrounding cartilage, cellularity, and necrosis were studied by microscopic analysis based on the O’Driscoll scale [62,89,90]. Microscopic evaluation was performed by a histopathologist blinded to group assignment.

#### 3.2.6. Statistical Evaluation

For the analysis of macroscopic and microscopic data, the mean, standard deviation (SD), and median were calculated. Comparisons between experimental groups were performed using the Mann–Whitney test, with a significance threshold set at 0.05.

## 4. Conclusions

The developed protocol for chondrocyte isolation and culture is sufficient to yield an adequate number of cells suitable for transplantation.

Elemental analysis of PES scaffold slices after 2 weeks of chondrocyte culture revealed a tissue concentration of 0.24 mg per membrane section.

Autologous chondrocyte implantation promoted the formation of regenerated tissue with a morphology resembling hyaline-like cartilage.

The quality of the tissue regenerated following cartilage cell transplantation onto the PES scaffold was comparable to that achieved with the PES scaffold alone, reached maturity by 12 weeks, and maintained a hyaline-like cartilage-like structure throughout the 52-week follow-up period.

A statistically significant difference was found between the number of points obtained in groups I and II in the shortest and medium observation periods, suggesting that regenerative outcomes in group II improved with longer follow-up.

## Figures and Tables

**Table 1:** Tissue mass content from PES scaffolds after 14 days of culture.

Number of PES Scaffold	Tissue Mass Content [mg]
1	0.29
2	0.19
3	0.26
4	0.22

**Table 2:** Distribution of summary of macroscopic assessment results, including degree of defect repair and macroscopic appearance in individual observation periods.

Observation Period	Group	Number of Knees with Rating				Sum of Points
		0	1	2	3	
12 weeks	I		1		7	8
	II	1	1	5	1	8
	III	2	2			4
25 weeks	I			2	6	8
	II	1	1	4	2	8
	III	2	2			4
52 weeks	I		1		8	9
	II		1	2	6	9
	III	1	3			4

**Table 3:** Assessment of summary results for macroscopic evaluation in groups I, II, and III after 12, 28, and 54 weeks.

Time	12 Weeks			25 Weeks			52 Weeks		
Group of rabbits	I	II	III	I	II	III	I	II	III
Average	6	4.2	1.2	6.2	4	1.7	6.2	5.6	2.2
SD	1.3	1.8	0.8	1.2	1.6	0.8	1.6	1.6	0.8
Median	6	4	1.5	6	4	1.5	6	6	2.5

**Table 4:** Comparison between groups I and II: summary of macroscopic assessment results, including degree of defect repair and macroscopic appearance (Mann–Whitney test).

Compared Groups	Statistical Significance of Differences Among Groups Shaded Fields Indicate Statistically Significant Results (*p* ≤ 0.05)		
	After 12 Weeks	After 25 Weeks	After 52 Weeks
I/II	0.046	0.021	0.427

**Table 5:** Results of Mann–Whitney test evaluating differences in overall assessment between observation periods.

Groups	Statistical Significance Level of Differences Between Observation Periods. Shaded Fields Indicate Statistically Significant Results (*p* ≤ 0.05)		
	12 Weeks → 25 Weeks	12 Weeks → 52 Weeks	25 Weeks → 52 Weeks
I	0.834	0.564	0.773
II	1.000	0.102	0.061

**Table 6:** Assessment of the sum of points for microscopic evaluation obtained in groups of rabbits I, II, and III after 12, 28, and 54 weeks.

Time	12 Weeks			25 Weeks			52 Weeks		
Group of rabbits	I	II	III	I	II	III	I	II	III
Average	10.5	8.3	5.2	13	12	4	14.2	14.3	4.5
SD	4.7	2.2	2.2	3.8	4.2	1.8	3.9	1.5	2
Median	11	8.5	5	14	13.5	4	15	14	4.5

**Table 7:** The distribution of the summary of microscopic assessment results, including the nature of predominant tissue and structural characteristics in individual observation periods.

Observation Period	Group	Number of Knees with Rating				Sum of Points
		0–6	7–10	11–14	15–18	
12 weeks	I	1	2	3	2	8
	II	2	4	2		8
	III	3	1			4
25 weeks	I	1	1	2	4	8
	II	1	1	5	1	8
	III	4				4
52 weeks	I	1		1	7	9
	II			5	4	9
	III	3	1			4

**Table 8:** Comparison between groups I and II: summary of microscopic assessment results, including nature of predominant tissue and structural characteristics (Mann–Whitney test).

Compared Groups	Statistical Significance of Differences Among Groups Shaded Fields Indicate Statistically Significant Results (*p* ≤ 0.05)		
	After 12 Weeks	After 25 Weeks	After 52 Weeks
I/II	0.141	0.564	0.331

**Table 9:** Results of the Mann–Whitney test evaluating differences in the overall assessment between observation periods.

Groups	Statistical Significance Level of Differences Between Observation Periods. Shaded Fields Indicate Statistically Significant Results (*p* ≤ 0.05)		
	2 Weeks → 25 Weeks	12 Weeks → 52 Weeks	25 Weeks → 52 Weeks
I	0.294	0.043	0.441
II	0.031	0.001	0.163

## Data Availability

The original contributions presented in this study are included in the article. Further inquiries can be directed to the corresponding author.
